# Salvaging hilar access using an uneven double-lumen cannula in endoscopic ultrasound-guided hepaticogastrostomy

**DOI:** 10.1055/a-2704-6653

**Published:** 2025-09-26

**Authors:** Ryosuke Sato, Kazuyuki Matsumoto, Akihiro Matsumi, Kazuya Miyamoto, Yuki Fujii, Daisuke Uchida, Motoyuki Otsuka

**Affiliations:** 1 92057 Department of Gastroenterology and Hepatology, Okayama University Hospital, Okayama, Japan

A common challenge in endoscopic ultrasound-guided hepaticogastrostomy (EUS-HGS) is misdirection of the guidewire into the peripheral bile duct instead of the desired hilar direction. This situation, which often occurs with acute puncture angles, complicates the procedure. The uneven double-lumen cannula (UDC) (Uneven Double Lumen Cannula; Piolax Medical, Kanagawa, Japan) is a valuable tool in these cases, enabling successful hilar guidewire placement as a rescue technique known as the “uneven method” 1 . UDCs have two lumen orifices a short (5-mm) or long (30-mm) distance apart. The long-type UDC is particularly useful for hilar guidewire access because its distal tip remains securely in the bile duct, allowing precise proximal orifice repositioning.

A 68-year-old woman who had undergone pancreaticoduodenectomy presented with liver dysfunction. Computed tomography revealed dilated bile ducts ( Fig. 1 ) consistent with cholangitis. An initial attempt at biliary stenting via double-balloon endoscopic retrograde cholangiopancreatography was unsuccessful because of jejunal obstruction caused by peritoneal metastasis. Therefore, EUS-HGS was performed for biliary stenting ( Video 1 ). Under EUS guidance (GF-UCT260; Olympus Medical, Tokyo, Japan), the B3 bile duct was punctured using a 22-gauge fine-needle aspiration needle (EZ Shot 3 Plus; Olympus Medical). However, attempts to advance a 0.018-inch guidewire (J-Wire Premier 18 NM; J-MIT, Kyoto, Japan) toward the hilum were unsuccessful, and the guidewire was consistently tracked into a peripheral branch ( Fig. 2 ). After peripheral positioning of the guidewire, a long-type UDC was inserted ( Fig. 3 ). A 0.025-inch guidewire (Visiglide2; Olympus Medical) was then successfully navigated into the hilum through the proximal lumen orifice of the UDC and across the hepaticojejunostomy anastomosis. The initial 0.018-inch guidewire was then re-inserted to establish a double-guidewire platform 2 ( Fig. 4 ). An uncovered self-expandable metallic stent (Yabusame Neo; Kaneka Medix, Osaka, Japan) was placed as antegrade stenting, and a 7-Fr plastic stent (Type IT; Gadelius Medical, Tokyo, Japan) was placed across the EUS-guided created route ( Fig. 5 ).

**Fig. 1:**
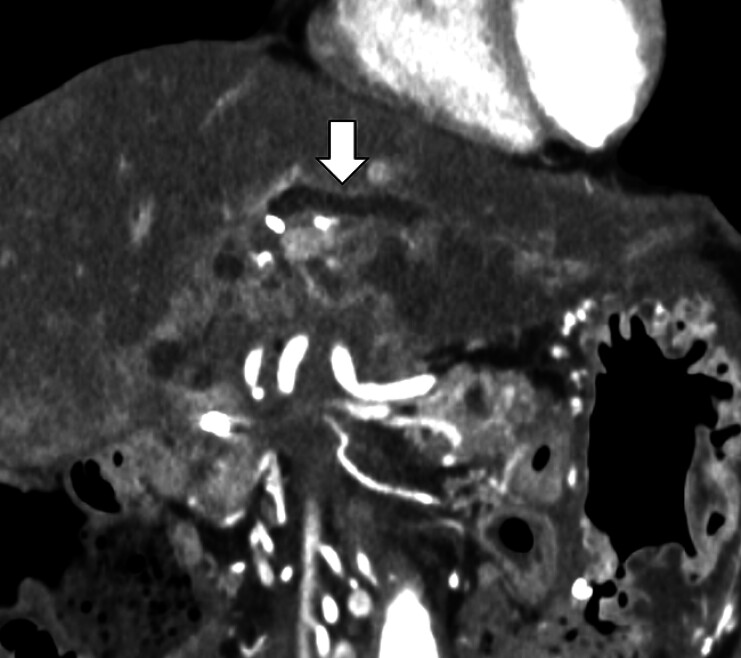
Computed tomography revealed dilated bile ducts (arrow), consistent with cholangitis after pancreaticoduodenectomy.

Salvage method for achieving successful hilar access using a long-type uneven double-lumen cannula when the guidewire has been misdirected into the peripheral bile duct.Video 1

**Fig. 2:**
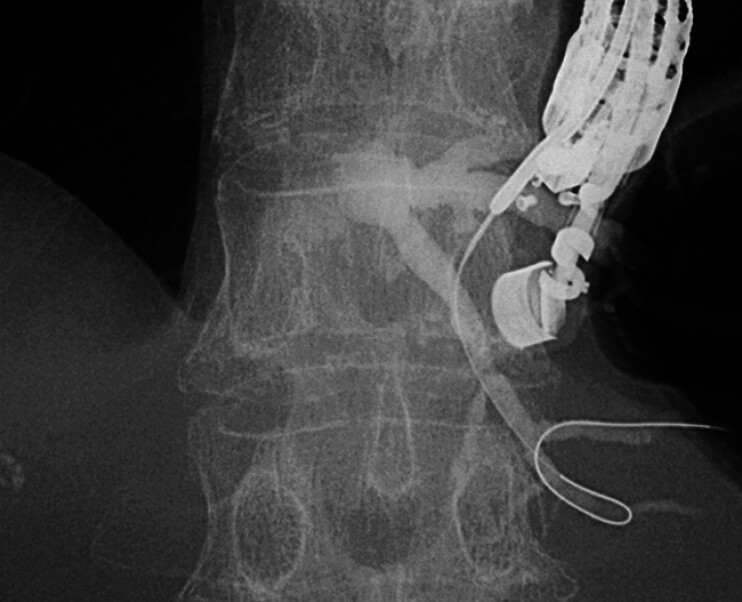
The B3 bile duct was punctured at an acute angle, causing the guidewire to advance only into the periphery.

**Fig. 3:**
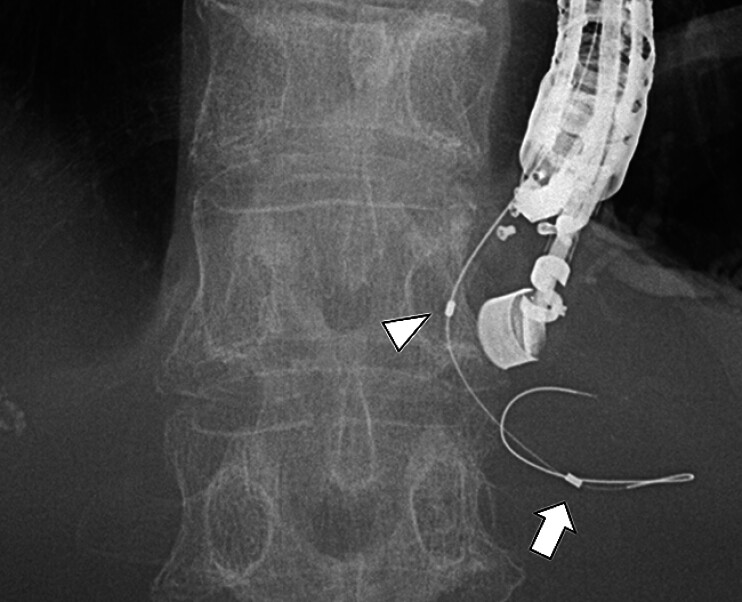
After peripheral positioning of the guidewire, a long-type uneven double-lumen cannula (UDC) was inserted. The distal tip of the UDC remained securely in the bile duct (arrow), and the proximal orifice of the UDC was positioned at the biliary duct puncture site in order to navigate a second guidewire into the hilum (arrowhead).

**Fig. 4:**
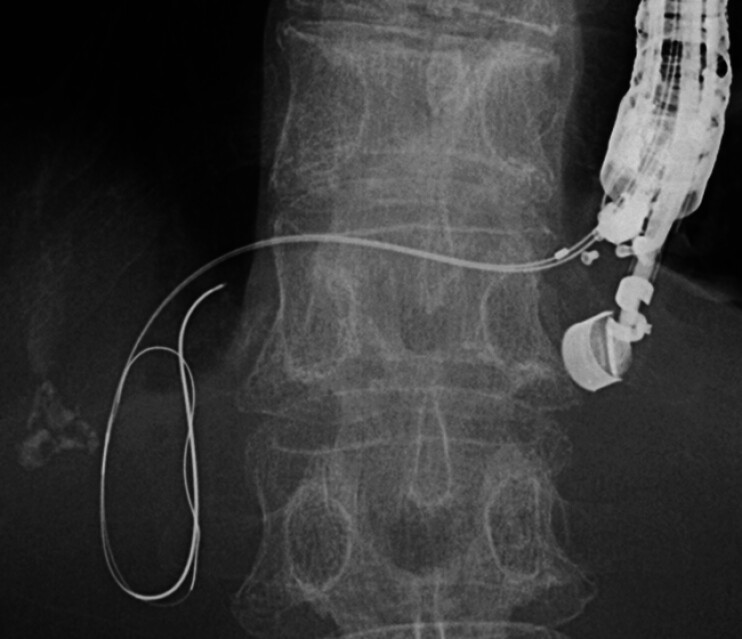
The second guidewire was successfully inserted into the hilum through the proximal orifice and across the hepaticojejunostomy anastomosis. The first guidewire was then reinserted to establish a double-guidewire platform.

**Fig. 5:**
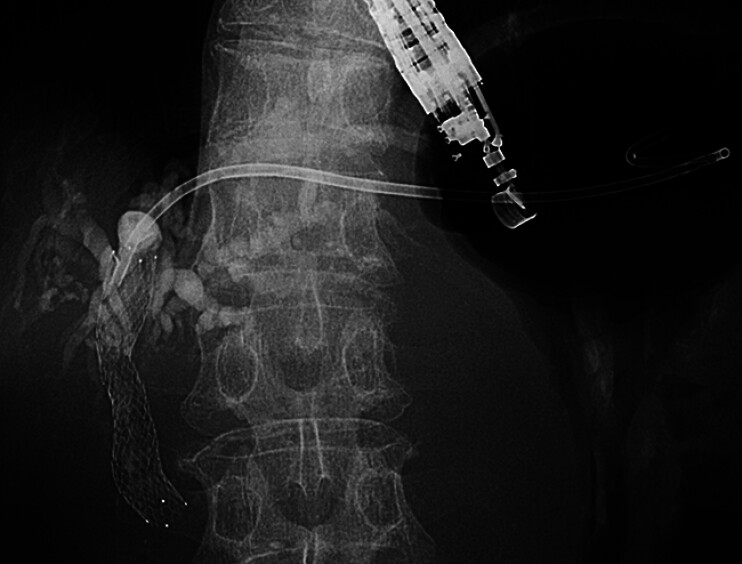
An uncovered self-expandable metallic stent was placed across the hepaticojejunostomy anastomosis as antegrade stenting, and a 7-Fr plastic stent was placed across the EUS-guided created route.

Endoscopy_UCTN_Code_CPL_1AL_2AD

## Notes

Endoscopy E-Videos https://eref.thieme.de/e-videos
*E-Videos* is an open access online section of the journal *Endoscopy* , reporting on interesting cases and new techniques in gastroenterological endoscopy. All papers include a high-quality video and are published with a Creative Commons CC-BY license. Endoscopy E-Videos qualify for HINARI discounts and waivers and eligibility is automatically checked during the submission process. We grant 100% waivers to articles whose corresponding authors are based in Group A countries and 50% waivers to those who are based in Group B countries as classified by Research4Life (see: https://www.research4life.org/access/eligibility/ ). This section has its own submission website at https://mc.manuscriptcentral.com/e-videos .
